# Epoetin alfa in platinum-treated ovarian cancer patients: results of a multinational, multicentre, randomised trial

**DOI:** 10.1038/sj.bjc.6603004

**Published:** 2006-03-28

**Authors:** P M Wilkinson, M Antonopoulos, M Lahousen, M Lind, P Kosmidis

**Affiliations:** 1 Christie Hospital, Wilmslow Road, Manchester M20 4BX, UK; 2 Athina Hospital, Athens, Greece; 3 University Klinik fur Gynakologie, Graz, Austria; 4 Hull Royal, Hull, UK; 5 Ygeia Hospital, Athens, Greece

**Keywords:** anaemia, epoetin alfa, haemoglobin, ovarian, quality of life

## Abstract

This multicentre, open-label, controlled clinical trial assessed the effects of epoetin alfa treatment on haematologic and quality of life (QOL) parameters in 182 anaemic (Hb⩽12 g dl^−1^) ovarian cancer patients receiving platinum chemotherapy. Patients were randomised 2 : 1 to receive epoetin alfa 10 000–20 000 IU three times weekly plus best standard treatment (BST) or BST only. Main study end points were changes from baseline in haemoglobin (Hb) level, transfusion requirements, and QOL. For the epoetin alfa group, mean Hb increased by 1.8 g dl^−1^ by weeks 4–6 and was significantly increased from baseline through study end (*P*<0.001). The mean change in Hb from baseline was significantly (*P*<0.001) greater for epoetin alfa than BST patients at all postbaseline evaluations. Significantly fewer epoetin alfa than BST patients required transfusion(s) after the first 4 weeks of treatment (7.9 *vs* 30.5%; *P*<0.001). Also, significant (*P*⩽0.04) differences favouring the epoetin alfa group over the BST group were found for all three median CLAS scores (Energy Level, Ability to Do Daily Activities, Overall QOL) and the median average CLAS score during chemotherapy. These findings support use of epoetin alfa to increase Hb levels, reduce transfusion use, and improve QOL in anaemic ovarian cancer patients receiving platinum chemotherapy.

Ovarian cancer is the fourth most common cancer in women and the second most common gynaecological cancer (Harries and Gore, 2002a, 2002b). Although the 5-year survival rate for women with low-risk stage I epithelial ovarian cancer can be as high as 90% (Memarzadeh and Berek, 2001), this form of cancer is frequently not detected until it is in its advanced stages with corresponding 5-year survival rates of 20–40 and 10% for stage III and stage IV disease, respectively (Heintz *et al*, 2001). Standard therapy for ovarian cancer is cytoreductive surgery, followed in most cases by platinum/paclitaxel combination chemotherapy (Harries and Gore, 2002a). Although chemotherapy has been shown to increase survival for women with ovarian cancer, most eventually relapse and die (Harries and Gore, 2002b). Therefore, palliating symptoms and maintaining quality of life (QOL) have become primary goals in disease management.

Anaemia in ovarian cancer patients, while often related to the disease itself, also commonly results from myelosuppression induced by repeated cycles of platinum-based chemotherapy. In a large-scale audit of patients in the United Kingdom receiving chemotherapy, which included 856 patients with ovarian cancer, the proportion of ovarian cancer patients with anaemia (haemoglobin (Hb) <11 g dl^−1^) rose from about 25% after chemotherapy cycle 1 to 50% after cycle 6, despite 41% of these patients having received at least one blood transfusion during treatment (Barrett-Lee *et al*, 2000). The most commonly administered chemotherapeutic agents administered in this subgroup were carboplatin (63%), and a combination of doxorubicin+cisplatin+cyclophosphamide (15%).

It is well known that severe anaemia is associated with an array of debilitating symptoms; however, even mild anaemia (Hb level 10–12 g dl^−1^) can have serious negative consequences for patients. An Hb level <12 g dl^−1^ has been associated with increased risk of transfusion (Ray-Coquard *et al*, 1999), increased fatigue (Cella, 1997; Holzner *et al*, 2002), and a less-than-optimal QOL (Crawford *et al*, 2002). Recently, important data on anemia and performance status were obtained by the European Cancer Anaemia Survey (ECAS). A prospective, multinational observational survey, ECAS evaluated the prevalence, incidence, and treatment of anaemia in 15 367 European cancer patients, 1741 of whom had gynaecologic malignancies (Ludwig *et al*, 2004). Analysis of the survey data showed that the prevalence and incidence of anaemia in the gynaecologic subgroup were 81.4 and 74.8%; however, despite its high prevalence and incidence, anaemia remained untreated in 57.3% of patients with this symptom (Barrett-Lee *et al*, 2005). The analysis additionally showed a significant correlation between low Hb levels and poor performance score, as assessed by WHO criteria (*P*<0.001, *R*=−0.18). From this it can be inferred that a substantial proportion of anemic gynaecologic cancer patients experience a decline in functional capacity, with a subsequent decline in QOL. Given the many negative consequences of anaemia for ovarian cancer patients, maintaining optimal Hb levels should be considered an essential aspect of supportive care.

Epoetin alfa therapy has been shown in both double-blind, placebo-controlled, and open-label studies to increase Hb levels in cancer patients receiving platinum- or nonplatinum-based chemotherapy, correcting anaemia, decreasing transfusion requirements, and subsequently improving patients’ QOL (Abels, 1992; Leitgeb *et al*, 1994; Glaspy *et al*, 1997; Demetri *et al*, 1998; Dammacco *et al*, 2001; Gabrilove *et al*, 2001; Littlewood *et al*, 2001; Thomas, 2002; Janinis *et al*, 2003; Shasha *et al*, 2003; Savonije *et al*, 2004; Chang *et al*, 2005; Witzig *et al*, 2005). However, few studies to date have examined the outcome of anaemia treatment in ovarian cancer patients, and none have specifically evaluated the impact of anaemia treatment on QOL in this population (ten Bokkel Huinink *et al*, 1998). Given the relative lack of data on anaemia treatment in ovarian cancer patients, we conducted a study to determine the possible benefits of epoetin alfa treatment with respect to transfusion reduction, QOL, and anaemia-related symptoms, including fatigue, in anemic ovarian cancer patients.

## MATERIALS AND METHODS

### Study patients and design

This was a phase IV, multinational, multicentre, randomised, open-label, comparative clinical trial conducted between March 1999 and June 2001. Female patients 18 years of age or older with a confirmed diagnosis of ovarian cancer, at least mild anaemia (Hb⩽12 g dl^−1^), and an ECOG performance score of 0, 1, 2, or 3 were enrolled. Patients were to be receiving or scheduled to receive platinum-based chemotherapy and were to have a life expectancy of at least 6 months. Patients with untreated iron, folate, or vitamin B_12_ deficiency or anaemia due to factors other than cancer or its treatment were excluded. Also excluded were those who had received a blood transfusion within 14 days prior to study entry or who had severe illness or surgery within 7 days of study entry. All patients provided written informed consent before study entry. The study was undertaken after approval of the protocol by the Independent Ethics Committee of each centre, and was conducted in accord with the Guidelines for Good Clinical Practice and the Declaration of Helsinki, South Africa amendment 1996.

Enrolled patients were randomised 2 : 1 to receive either epoetin alfa plus best standard treatment (BST; transfusion of red blood cells, as needed) or BST only. Outside of the United States, epoetin alfa is manufactured by Ortho Biologics, LLC, and distributed and marketed as EPREX® or ERYPO® by Ortho Biotech, a division of Janssen-Cilag. Epoetin alfa was administered initially at a dosage of 10 000 IU three times weekly (t.i.w.) subcutaneously (or 5000 IU t.i.w. for patients with body weight <45 kg). The initial dose was maintained throughout the first cycle of a 4-week cycle of chemotherapy or the first two cycles of a three-week cycle. If, at the end of the initial period, the reticulocyte count had not increased by >40 000 *μ*l^−1^, or Hb had not increased by >1 g dl^−1^ above the baseline level, the dose of epoetin alfa was doubled (maximum allowed dosage, 20 000 IU t.i.w.). If the Hb level exceeded 14 g dl^−1^ at any time, study drug was withheld until the Hb level had declined to <12 g dl^−1^ and was then restarted at a dose 25–50% lower than the previous dose. If the Hb level increased by ⩾2 g dl^−1^ month^−1^, the dose was reduced by 25–50% to maintain the Hb rate of increase at <2 g dl^−1^ month^−1^. Dosage reductions could be achieved by omitting one of the weekly doses of epoetin alfa. No adjustment to the dosage was made if Hb level increased in response to transfusion. The planned duration of study treatment was a maximum of 28 weeks, which included 18–24 weeks of chemotherapy (maximum, six cycles) plus up to 4 weeks after the last chemotherapy dose.

In both arms, red blood cell transfusion was permitted during the study if judged necessary, but physicians were asked to refrain from transfusing patients unless the Hb level was <9 g dl^−1^. Administration of white cell growth factor was permitted, and a daily dose of 200-mg elemental iron as oral iron supplementation was recommended to prevent restriction of erythropoiesis. Transferrin saturation ⩽20% was considered indicative of inadequate iron stores and iron deficiency.

### Efficacy and safety evaluations

The primary efficacy end point was the difference between the treatment groups in change in Hb level from baseline to study end. Secondary efficacy end points included within-group change in Hb level from baseline to study end and between-group differences in proportions of patients considered complete responders, partial responders, or nonresponders. Complete responders were defined as patients who demonstrated an Hb increase ⩾1 g dl^−1^ above baseline without transfusion within the preceding 4 weeks. Partial responders were defined as patients who achieved an Hb increase of ⩾0.5 g dl^−1^ but <1 g dl^−1^, and nonresponders, as those who either were transfused or demonstrated an Hb increase of <0.5 g dl^−1^ above baseline.

Other efficacy end points included change in proportion of patients transfused and change in QOL scores from baseline to study completion. Quality of life was assessed using the patient-rated Cancer Linear Analog Scale (CLAS, also known as the Linear Analog Scale Assessment (LASA)), which measures Energy Level, Ability to Do Daily Activities, and Overall QOL, and the Functional Assessment of Cancer Therapy-Anaemia (FACT-An), for which the FACT-General (FACT-G Total) scale, FACT-An Fatigue subscale, and Nonfatigue subscale were assessed. Both the FACT-An and the CLAS scales are cancer specific and sensitive to Hb levels (Cella, 1997; Glaspy *et al*, 1997; Demetri *et al*, 1998; Gabrilove *et al*, 2001). Haemoglobin levels, transfusion data, and QOL scores were obtained within 7 days prior to the first dose of study medication and at study completion or early termination. During the study, Hb levels were measured and transfusion data collected on completion of study weeks 4 or 6, 8 or 9, 12, and 16 or 18; QOL was assessed after 4 or 6 weeks (CLAS only), 8 or 9 weeks (CLAS and FACT-An), and 12 weeks (CLAS only). Additionally, tumour response to chemotherapy and/or radiotherapy was assessed at study end or the final visit.

Safety and tolerance of epoetin alfa were evaluated by the usual methods, including monitoring adverse events. Adverse events were reported by patients throughout the study either spontaneously or in response to general, nondirect questioning by the investigator.

### Statistical analyses

The primary analysis was based on the intent to treat (ITT) population. For efficacy evaluations, changes between baseline and each monthly value for Hb level were analysed using both analysis of variance (ANOVA) and the Wilcoxon rank-sum test. The proportion of patients transfused during the treatment period was analysed by Fisher's exact test, changes from baseline in QOL scores were analysed using the Wilcoxon signed rank-sum test, and tumour stage was compared using the Wilcoxon rank-sum test. All *P*-values were unadjusted and were derived from two-sided tests. A *P*-value of ⩽0.05 was considered to indicate statistical significance.

A total of 145 evaluable patients were required to complete the study to have a 90% power to detect a difference (2.0 g dl^−1^) between the epoetin alfa and BST groups in change in Hb from baseline to last evaluation (primary variable), with randomisation assignment to one of two treatment arms in a 2 : 1 ratio. Tests of significance were one- or two-sided, with *α* set at 0.05 or 0.025. The study was not powered for secondary efficacy variables, including QOL.

## RESULTS

In total, 182 patients were enrolled in the study, 173 (114 epoetin alfa; 59 BST) of whom were eligible for efficacy evaluation (ITT population). The nine ineligible patients were excluded because of data recording on differently designed case report forms (4), misdiagnosis (3), or withdrawal of patient consent (2). The majority (91) of evaluable patients were seen at centers in the United Kingdom; the remaining patients were treated at centers in Austria (27), Greece (26), Sweden (22), The Netherlands (5), and Denmark (2). Of the 173 patients, 145 completed the study. The other 28 were discontinued prematurely for the following reasons: adverse event, insufficient response, noncompliance, asymptomatic/cure, disease progression, chemotherapy discontinued, and cancer not ovarian. Three of the 28 patients, all in the epoetin alfa group, died during the study as a result of their malignancy. All three patients were included in the study despite having a life expectancy of <6 months at entry, and their deaths were not unexpected.

Of 119 patients with available drug exposure information, 117 commenced dosing with 30 000 IU week^−1^, whereas two patients commenced dosing with 20 000 and 27 000 IU week^−1^, respectively. (The two latter patients violated the protocol requirement for an initial starting dose of 30 000 IU week^−1^, but this was considered a minor violation and the patients were therefore included for analysis.) In total, 10 patients (8%) required dose increases during the study.

Baseline demographic and clinical characteristics were generally comparable between the two treatment groups (Table 1). Mean Hb levels at baseline were 10.8 g dl^−1^ for patients given epoetin alfa and 10.7 g dl^−1^ for those given BST. More than 90% of the patients in each group were receiving carboplatin or carboplatin plus paclitaxel, and the remainder were receiving cisplatin. The frequencies of 3- and 4-week chemotherapy cycles also were comparable between the two groups (3-week cycles: epoetin alfa, 73.7%; BST, 72.9%) (4-week cycles: epoetin alfa, 26.3%; BST, 27.1%).

### Haematopoietic response

**Figure 1:**
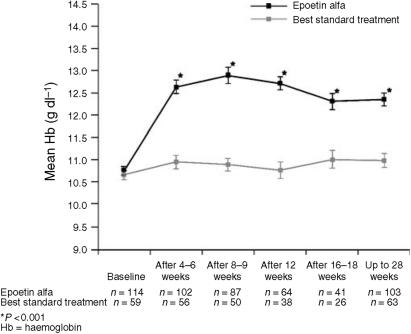
Mean haemoglobin levels±s.e. over time (epoetin alfa *vs* best standard treatment).

The evaluation of haematopoietic response was based on the 171 patients who received uninterrupted treatment. In the epoetin alfa group, the Hb level increased by a mean of 1.8 g dl^−1^ after the first 4–6 weeks of treatment, and was significantly (*P*<0.001) increased above baseline at all time points (Figure 1). In contrast, Hb levels in the BST group changed little over the course of treatment. Differences between the epoetin alfa and BST groups were significant (*P*<0.001, ANOVA or *t*-test) at all post-baseline evaluations. The highest Hb levels in the epoetin alfa group were observed after weeks 8–9 and 12. Mean±s.d. increases in Hb level from baseline after 8–9 weeks were 2.0±1.5 g dl^−1^ for the epoetin alfa group *vs* 0.3±1.0 g dl^−1^ for the BST group; corresponding values after 12 weeks were 1.8±1.3 *vs* 0.0±1.1 g dl^−1^. At study completion or early termination, mean changes in Hb level from baseline were 1.6±1.5 and 0.3±1.3 g dl^−1^, respectively, for the epoetin alfa and BST groups.

**Figure 2:**
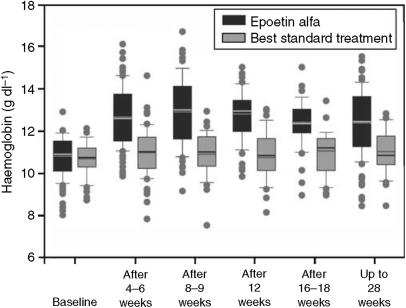
Box and whisker plots summarising the distribution of haemoglobin values for all patients in the two study groups during the treatment period. Bottom and top edges of the boxes correspond to the 25th and 75th percentiles of the sample, respectively. The central solid lines represent the median values, and the dotted lines represent the mean values. The bars at the ends of the vertical lines or ‘whiskers’ mark the 5 and 95% values. Any values more extreme than these are indicated by solid black circles.

The distribution of patients in the two treatment groups by Hb level during treatment is illustrated in Figure 2. As shown, patients treated with epoetin alfa above the 25th percentile had Hb values higher than those for BST patients in the 75th percentile at weeks 8–9 and week 12, and in the 95th percentile after weeks 16–18. Median (interquartile range) Hb levels for the epoetin alfa and BST groups were 13.0 (2.5) *vs* 11.0 (1.4) g dl^−1^, respectively, after 8–9 weeks, and 12.9 (1.5) g dl^−1^ *vs* 10.8 (1.5) g dl^−1^, respectively, after 12 weeks. Overall, more patients in the epoetin alfa group than in the BST group had Hb increased by ⩾1 g dl^−1^ (responders, 78 *vs* 32% for epoetin alfa and BST, respectively; Table 2). Conversely, fewer patients in the epoetin alfa group did not respond to treatment. The difference in the proportions of both responders and nonresponders between the treatment groups was statistically significant (*P*<0.001).

### Transfusion use

A significantly smaller proportion of patients in the epoetin alfa group (nine out of 114, or 7.9%) than in the BST group (18 out of 59, or 30.5%) were transfused at least once after the first 4 weeks of treatment (*P*<0.001, Fisher's exact test). Also, significant differences in transfusion rate favouring epoetin alfa were noted at all evaluations except week 12, at which time the difference favoured epoetin alfa, but not significantly: week 4 or 6, 5.9 *vs* 16.1%, *P*=0.048; week 8 or 9, 0.0 *vs* 14.0%, *P*<0.001; week 12 : 1.6 *vs* 5.3%, not significant; weeks 16–18, 0.0 *vs* 19.2%, *P*=0.007; and up to 28 weeks: 1.8 *vs* 13.8%, *P*=0.004.

### Quality of life

**Figure 3:**
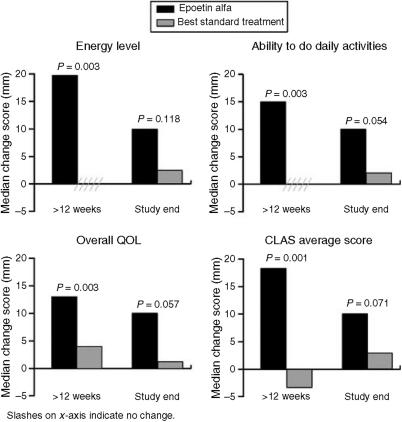
Change in Cancer Linear Analog Scale (CLAS, also known as Linear Analog. Scale Assessment (LASA)) scores from baseline after 12 weeks and at study end (epoetin alfa *vs* best standard treatment).

Of the 173 evaluable patients, 102 (64 epoetin alfa, 38 BST) had paired CLAS data for baseline and after 12 weeks, and 141 (91 epoetin alfa, 50 BST) had such data for baseline and end of study. Analysis of these data (Figure 3) showed significant differences from baseline favouring epoetin alfa over BST for all three CLAS change scores (Energy Level, Ability to Do Daily Activities, Overall QOL) and the average median CLAS change score during chemotherapy (*P*⩽0.04; after 12 weeks: *P*⩽0.003; Wilcoxon signed rank-sum test). At the final visit, the median increase was 10.0 mm for each parameter in the epoetin alfa group compared with median increases of 1.25–2.85 mm for these parameters in the BST group; differences between the groups did not achieve statistical significance (*P=*0.054–0.118). Results of within-treatment analysis showed that median scores for each of the three CLAS scales and the average median CLAS score were significantly increased from baseline at all four evaluation points in the epoetin alfa group (*P*⩽0.001, Wilcoxon signed rank test), whereas no significant change from baseline was detected at any evaluation point in the BST group. In the epoetin alfa group, the average median CLAS score increased by 36% from baseline (56.85 mm) to last observation (77.30 mm), with increases of up to 45% (to 82.70 mm) noted after 12 weeks. In contrast, the BST group showed little change in average median score from baseline (62.00 mm) to last observation (70.00 mm) or to any evaluation point during chemotherapy (maximum, 63.30 mm after 8 or 9 weeks).

The study was underpowered for FACT-An analysis. However, univariate analysis demonstrated trends favouring epoetin alfa over BST for the FACT-An Nonfatigue score (*P=*0.087) after 8–9 weeks and for the FACT-G Total (*P=*0.081), FACT-An Fatigue (*P=*0.173), and FACT-An Nonfatigue (*P=*0.082) subscale scores at study end.

### Tumour response

At the end of treatment, the two study groups were similar with respect to the proportions of patients in each group with complete response, partial response, or no response to cancer treatment (Table 3). However, proportionally more patients in the epoetin alfa group than in the BST group had progressive disease, although this difference was not significant (11 *vs* 2%, *P*=0.425). Examination of the patients’ medical histories showed that the status of progressive disease was related to disease stage at study entry.

### Safety

Of the 182 patients enrolled, 181 (121 epoetin alfa, 60 BST) received study treatment and were included in the safety analysis. The overall incidence of adverse events was similar between the two treatment groups; 74% of patients given epoetin alfa and 73% of those given BST reported at least one adverse event. The most common adverse events involved the gastrointestinal tract; these included nausea (epoetin alfa: 18%; BST: 20%), constipation (epoetin alfa: 15%; BST: 10%), and vomiting (epoetin alfa: 10%; BST: 8%). A total of 12 thrombotic vascular events (TVEs) were reported by 10 patients (8.3%) receiving epoetin alfa, including deep vein thrombosis (three events), pulmonary embolus (three events: two in one patient, one in another patient), cerebrovascular accident (two events), thrombosis (two events), thrombophlebitis (one event), and weakness on left or right side (one event); one TVE (thrombophlebitis) was reported in one BST patient (1.7%). Hypertension was reported in three epoetin alfa-treated patients (mild – 1, moderate – 2). Serious adverse events (SAEs) were reported in 23% of patients given epoetin alfa and 15% given BST. In the epoetin alfa group, the most common SAEs were deep vein thrombosis (three patients), and ascites, pain, vomiting, pulmonary embolism, fracture, and sepsis (two patients each). In the BST group, the most common SAEs were ascites and anemia (two patients each). All other SAEs occurred in one patient each. Three patients, all in the epoetin alfa group, died during the study, none because of an adverse event related to study drug. Also, no patient died because of a TVE, and all patients with TVEs recovered except for one individual in the epoetin alfa group whose thrombophlebitis had not yet resolved at the time of data analysis. A total of 17 patients in the epoetin alfa group but no patient in the BST group discontinued prematurely from the study because of adverse events.

## DISCUSSION

The results of our study show that epoetin alfa can effectively increase Hb levels and reduce transfusion use for ovarian cancer patients receiving platinum-based chemotherapy. The mean Hb levels for the epoetin alfa group were significantly increased from baseline at all evaluations (*P*<0.001) and 78% of patients who received epoetin alfa demonstrated a complete response to anaemia treatment (Hb increase ⩾1 g dl^−1^ without transfusion within the previous 4 weeks). Response was rapid, with a 1.8 g dl^−1^ increase in mean Hb level at 4–6 weeks, and a maximum increase of 2.0 g dl^−1^ at 8–9 weeks. This rate of Hb increase compares favourably to rates seen in previous studies in patients with gynaecologic or other tumour types. In two large studies with patients receiving platinum or nonplatinum chemotherapy, mean Hb increases were about 1 g dl^−1^ at week 4 and 2 g dl^−1^ at week 8 (Gabrilove *et al*, 2001; Littlewood *et al*, 2001). In a study of epoetin alfa in patients with gynaecologic malignancies receiving polychemotherapy (70% with epithelial ovarian cancer), mean Hb increases of 1.5 and 1.6 g dl^−1^ were observed at 4 and 6 weeks, respectively, with an overall mean Hb increase of 3 g dl^−1^ at 12 weeks (Kurz *et al*, 1997). The differences in rates of Hb increase may reflect differences in baseline Hb for the populations in the various studies; the mean baseline Hb of 10.7 g dl^−1^ for ovarian cancer patients in our study was somewhat higher than that for patients in two of the other studies (range: 9.5–9.9 g dl^−1^).

Although safer than in the past, blood transfusion is still associated with numerous risks as well as substantial inconvenience for patients (Mohandas and Aledort, 1995), suggesting the need for alternative anaemia therapy. For epoetin alfa-treated patients in our study, increased Hb levels resulted in reduced transfusion use; only 7.9% of these patients received transfusions compared with 30.5% of BST patients (*P*<0.001) – a 74% reduction in transfusion use. This result is comparable to results from previous studies enrolling gynaecologic and ovarian cancer patients in which epoetin treatment reduced the percentage of patients requiring transfusion by 67–77% compared with controls (Kurz *et al*, 1997; ten Bokkel Huinink *et al*, 1998). In studies in patients with other malignancies, epoetin alfa treatment resulted in reductions of approximately 36–62% in transfusion use, compared with controls (Dammacco *et al*, 2001; Littlewood *et al*, 2001; Pronzato *et al*, 2002; Janinis *et al*, 2003; Savonije *et al*, 2004; Chang *et al*, 2005; Witzig *et al*, 2005) (Table 4).

The recognised negative impact of anaemia on cancer patient QOL further strengthens the need for correcting this condition, and, indeed, a number of large studies in cancer patients with a variety of solid tumours or nonmyeloid haematologic malignancies receiving chemotherapy have shown a positive relationship between increases in Hb level and improvements in QOL (Abels, 1992; Leitgeb *et al*, 1994; Glaspy *et al*, 1997; Demetri *et al*, 1998; Gabrilove *et al*, 2001; Littlewood *et al*, 2001; Shasha *et al*, 2003). However, only limited data relating specifically to QOL in gynaecologic cancer are available, particularly regarding the effects of anaemia and its treatment. Analysis of a subpopulation of gynaecologic patients from a large nonrandomised, open-label, community-based study of epoetin alfa in patients receiving chemotherapy showed a significant (*P*⩽0.01) correlation between increases in Hb level and improvement in QOL (Demetri, 1999). Mean change in Hb exceeded 2.0 g dl^−1^ over the 4-month study period, and overall transfusions were reduced from 36 to 6% (*P*<0.001). Mean increases in QOL from baseline to final score were significant (*P*<0.001) for Energy Level, Ability to Do Daily Activities, and Overall Quality of Life. In a small double-blind, placebo-controlled trial (*n*=35) that enrolled anaemic (Hb <11 g dl^−1^) gynaecologic patients receiving polychemotherapy, epoetin alfa treatment increased Hb levels by 3 g dl^−1^ at study end and significantly (*P*=0.009) decreased transfusion use (Kurz *et al*, 1997). Quality of life, measured using a nonvalidated questionnaire, did not differ significantly between the groups; however, physical activity levels improved significantly from baseline in patients who responded to epoetin alfa.

Our study was not powered to measure absolute change in QOL, but rather, statistical trends. Nevertheless, analysis of the QOL data showed that patients in the epoetin alfa group achieved significantly (*P*⩽0.04) greater improvement than patients in the BST group in all three median CLAS scores and in the average median CLAS score during chemotherapy. Moreover, average median CLAS scores for the epoetin alfa group were indicative of significant improvement at all evaluations, whereas those for the BST group after 12 weeks of treatment indicated a decline in QOL. Results for the FACT analyses showed trends favouring epoetin alfa over BST for the FACT-G and FACT-An subscale scores.

Other data indicative of a QOL benefit with epoetin alfa treatment are provided by several randomised, controlled and single-arm studies in cancer patients with a variety of malignancies. Results of a meta-analysis involving 11 459 patients from 23 trials showed that epoetin alfa therapy significantly improved CLAS (20–25%), FACT-Fatigue (17%), and FACT-An (12%) scores from baseline (*P*=0.05), whereas scores for control groups were relatively unchanged or worsened (Jones *et al*, 2004). Of interest, one of these trials, a randomised, double-blind controlled study (Littlewood *et al*, 2001), showed that although a significantly greater proportion of placebo-treated patients than epoetin alfa-treated patients were transfused, the mean Hb level of the placebo patients was unchanged from baseline over the course of the study and their QOL did not improve, but rather, worsened. In contrast, epoetin alfa-treated patients demonstrated a significant increase in Hb level from baseline, and showed significant improvement in QOL domains.

The incidence of adverse events was similar between the epoetin alfa and BST groups in this study, although patients in the epoetin alfa group had a higher incidence of TVEs (12 events *vs* one event; incidence 8.3 *vs* 1.7%). These findings are consistent with those of other randomised studies in which patients received epoetin alfa administered as either a t.i.w. (Dammacco *et al*, 2001; Littlewood *et al*, 2001) or once-weekly (q.w.) (Chang *et al*, 2005; Witzig *et al*, 2005) regimen. The overall incidence of adverse events in these studies ranged from 73 to 88% for patients in the epoetin alfa groups and from 75 to 86% for those in the control groups. Also, the incidence of TVEs was comparable to that reported in our study, ranging from 5 to 10.8% for the epoetin alfa groups and 3 to 7.9% for the control groups. The Hb level required for study enrollment/randomisation and baseline Hb in these studies are shown in Table 4. That all erythropoiesis stimulating agents may increase the risk for TVE development is well established and this information is in the agents’ product labelling. It must be mentioned in this regard, however, that the results of two recently reported studies have suggested an adverse impact on survival conferred by erythropoiesis stimulating agents (Henke *et al*, 2003; Leyland-Jones, 2003), and that in one of the studies, increased mortality was considered partly attributable to TVEs (Leyland-Jones, 2003). Both studies included cancer patients who would not normally receive erythropoiesis stimulating agents, namely, nonanemic patients, and patients with Hb levels higher than those recommended in the approved labelling. Interpretation of the results of these studies is complicated due to the study designs and imbalance of risk factors in the populations. In contrast, a meta-analysis of 27 randomised, controlled studies (*N*=3287) of recombinant human erythropoietin (RHuEPO) showed that the relative risk for thromboembolic complications after RHuEPO treatment was not significantly increased compared with that of untreated patients (RR=1.58, 95% CI=0.94–2.66; 12 trials, *N*=1738). The absolute risk difference was 0.02 (95% CI 0.00–0.04), although the number of patients with no thrombotic event may have been underreported (Bohlius *et al*, 2005). Further, there was evidence of a trend toward improved overall survival with RHuEPO treatment. From the preceding, it can be concluded that epoetin alfa is safe and well tolerated when administered according to labelling, and that the risk of TVE development in cancer patients receiving epoetin therapy may be substantially limited by targeting the Hb concentration to around 12 g dl^−1^.

The natural history of ovarian cancer poses a unique challenge to anaemia management. In the majority of patients, this cancer is not diagnosed until it has reached more advanced stages. Although patients with advanced ovarian cancer typically respond well to first-line chemotherapy, most relapse and become candidates for further chemotherapy (Harries and Gore, 2002b). Prolonged disease, surgery, and repeated cycles of platinum-based or other chemotherapy all contribute to the development of anaemia in this population. Anaemia has been demonstrated to negatively affect cancer patients by increasing risk of transfusion and reducing QOL. The results of the present study support the use of epoetin alfa in anemic ovarian cancer patients to achieve a reliable haematologic response that is maintained throughout treatment. Evidence from this study further supports increasing Hb levels to ameliorate anaemia as a means of improving QOL – a primary goal of treatment for ovarian cancer patients with advanced disease and disease- or treatment-related anemia.

## Figures and Tables

**Table 1:** Demographic and clinical characteristics at baseline (intent-to-treat population, *N=*173)

**Characteristic**	**Epoetin alfa (*n=*114)**	**Best standard treatment (*n=*59)**
Mean age, year (±s.d.)	59.1 (±10.6)	60.3 (±11.2)
Range	35.0–87.0	30.0–79.0
Mean Hb level±s.d. (g dl^−1^)	10.75±0.94	10.66±0.83
		
*ECOG performance score* (*n, %)*^a^		
0	56 (49.1)	27 (45.8)
1	47 (41.2)	29 (49.2)
2	11 (9.6)	3 (5.1)
3	0 (0.0)	0 (0.0)
4	0 (0.0)	0 (0.0)
		
*Tumour stage, n* (*%)*		
I	12 (10.5)	10 (16.9)
II	13 (11.4)	3 (5.1)
III	58 (50.9)	32 (54.2)
IV	28 (24.6)	13 (22.0)
Unknown	3 (2.6)	1 (1.7)
		
*Metastatic disease, n* (*%)*		
Unknown	1 (0.9)	0 (0.0)
None	27 (23.7)	13 (22.0)
Abdominal	66 (57.9)	32 (54.2)
Liver	12 (10.5)	6 (10.2)
Lymphatic	12 (10.5)	6 (10.2)
Lung	7 (6.1)	5 (8.5)
Other type	34 (29.8)	17 (28.8)
		
*Previous surgery, n (%)*	94 (82.5)	49 (83.1)
		
*Type of chemotherapy, n* (*%)*		
Carboplatin	75 (65.8)	40 (67.8)
Carboplatin + paclitaxel	29 (25.4)	14 (23.7)
Cisplatin	10 (8.8)	5 (8.5)

a 0=able to carry out normal activities, 1=restricted physical activity/ambulatory/light work, 2=ambulatory/capable of all self-care/unable to work, 3=capable of only limited self-care, 4=completely disabled.

**Table 2:** Haematologic response by treatment group

**Haematologic response^a^**	**Epoetin alfa (*n=*112)**	**Best standard treatment (*n=*59)**
Complete responders, *n* (%)	87 (77.7)	19 (32.2)
Partial responders, *n* (%)	4 (3.6)	6 (10.2)
Nonresponders, *n* (%)	21 (18.8)	34 (57.6)

a Complete: Hb increase of ⩾1.0 g dl^−1^ above baseline without transfusion within previous 4 weeks; partial: Hb increase of ⩾0.5 g dl^−1^ but <1.0 g dl^−1^; no response: need for transfusion within previous 4 weeks or Hb increase of <0.5 g dl^−1^ above baseline. The difference in the proportions of both responders and nonresponders between the two treatment groups was statistically significant (*P*<0.001).

**Table 3:** Tumour response to cancer treatment

**Tumour response**	**Epoetin alfa (*n=*114)**	**Best standard treatment (*n=*59)**
Complete, *n* (%)	55 (48)	33 (56)
Partial, *n* (%)	25 (22)	19 (32)
None, *n* (%)	7 (6)	5 (9)
Progressive disease, *n* (%)	13 (11)	1 (2)
Unknown, *n* (%)	14 (12)	1 (2)

**Table 4:** Effect of epoetin alfa on transfusion: results of eight randomised, controlled studies

**Authors**	**Type study**	**Treatment/initial dose**	**Hb level criteria**	**Baseline Hb level**	**Transfusion requirements after Tx**
Kurz *et al* (1997)	R, DB, C (PBO) *N=*35 (gynaecologic)	EPO*α* 150 IU kg^−1^ t.i.w. or PBO	<11 g dl^−1^ (for inclusion)	*Mean:* EPO*α*: 9.9 g dl^−1^ PBO: 9.8 g dl^−1^	EPO*α*: 21.7% PBO: 66.6% (*P*=0.009)
					
Dammacco *et al* (2001)	R, DB with OL phase, C (PBO) *N=*145 (multiple myeloma)	EPO*α* 150 IU kg^−1^ t.i.w. or PBO	<11 g dl^−1^ (for inclusion)	*Mean:* EPO*α*: 9.3 g dl^−1^ PBO: 9.6 g dl^−1^	EPO*α*: 28% PBO: 47% (*P*=0.017)
					
Littlewood *et al* (2001)	R, DB, C (PBO) *N=*375 (mixed)	EPO*α* 150 IU kg^−1^ t.i.w. or PBO	⩽10.5 g dl^−1^ or >10.5–⩽12.0 g dl^−1^ after Hb↓ ⩾1.5 g dl^−1^ per chemo cycle (for enrollment)	*Mean:* EPO*α*: 9.9 g dl^−1^ PBO: 9.7 g dl^−1^	Total population: EPO*α*: 24.7% PBO: 39.5% (*P*=0.0057) EPO*α* stratum: >10.5 g dl^−1^: 7.1% ⩽10.5 g dl^−1^: 28.2%
					
Pronzato *et al* (2002)	R, OL, C (BST) *N=*178 (interim analysis) (breast cancer)	EPO*α* 10 000 IU t.i.w. or BST	10–12 g dl^−1^ (for initiation of treatment)	*Mean:* EPO*α*: 10.7 g dl^−1^ BST: 10.8 g dl^−1^	EPO*α*: 6.7% BST: 16.9% (*P*=0.06)
					
Janinis *et al* (2003)	R, OL, C (iron only) *N=*372 evaluable (mixed)	EPO*α* 10 000 IU t.i.w. + iron or iron only	⩽11 g dl^−1^ (for randomisation)	*Median:* 10.5 g dl^−1^ (both groups)	EPO*α* + iron: 9% Iron only: 23% (*P* < 0.0001)
					
Savonije *et al* (2004)	R, C (BSC) *N=*315 (mixed)	EPO*α* 10 000 IU t.i.w. or BSC	<12.1 g dl^−1^ (for randomisation)	*Median:* EPO*α*: 10.7 g dl^−1^ BSC: 10.8 g dl^−1^	EPO*α*: 37% BST: 65% (*P*<0.05)
Chang *et al* (2005)	R, OL, C (SOC) *N=*354 (breast cancer)	EPO*α* 40 000 IU q.w. or SOC	⩽15 g dl^−1^ (for entry) ⩽12 g dl^−1^ (for randomisation)	*Mean:* EPO*α*: 11.2 g dl^−1^ SOC: 11.3 g dl^−1^	EPO*α*: 8.6% SOC: 22.9% (*P*<0.001)
					
Witzig *et al* (2005)	R, DB, C (PBO) *N=*344 (mixed)	EPO*α* 40 000 IU q.w. or PBO	<11.5 g dl^−1^ (males) <10.5 g dl^−1^ (females) (for enrollment)	*Mean:* EPO*α*: 9.5 g dl^−1^ PBO: 9.4 g dl^−1^	EPO*α*: 25.3% PBO: 39.6% (*P*=0.005)

BSC=best standard care, BST=best standard treatment, C=controlled, DB=double-blind, EPO*α*=epoetin alfa, Hb=hemoglobin, NA=not available, NHP=Nottingham Health Profile, NS=not statistically significant, OL=open-label, PBO=placebo, q.w.=once weekly, R=randomised; SOC=standard of care, t.i.w.=3 times a week, Tx=treatment.

## Appendix

The following investigators participated in this study: Dr K Joerg, Villach, Austria; Dr H Hausmaninger, Salzburg, Austria; Dr M Lahousen, Graz, Austria; Dr J Maier, Wels, Austria; Dr MR Mirza, Odense, Denmark; Dr M Antonopoulos, Athens, Greece; Dr P Kosmidis, Athens, Greece; Dr P Paraskevopoulos, Thessaloniki, Greece; Dr Hdankbaar, Hengelo, The Netherlands; Dr HP Sleeboom, Den Haag, The Netherlands; Dr P Willemse, Groningen, The Netherlands; Dr H Andersson, Göteborg, Sweden; Dr H Malmstrom, Linkoping, Sweden; Dr M Ridderheim, Lund, Sweden; Dr J Davis, Glasgow, UK; Dr A Hong, Wonford, UK; Dr CJ Irwin, Coventry, UK; Professor M Lind, Hull, UK; Dr P Murray, Colchester, UK; Dr SD Pledge, Sheffield, UK; Professor N Reed, Glasgow, UK; Dr H Thomas, Guildford, UK; Dr PM Wilkinson, Manchester, UK.
